# Emotion Transfer, Emotion Regulation, and Empathy-Related Processes in Physician-Patient Interactions and Their Association With Physician Well-Being: A Theoretical Model

**DOI:** 10.3389/fpsyt.2018.00389

**Published:** 2018-08-28

**Authors:** Sonja Weilenmann, Ulrich Schnyder, Brian Parkinson, Claudio Corda, Roland von Känel, Monique C. Pfaltz

**Affiliations:** ^1^Department of Consultation-Liaison Psychiatry and Psychosomatic Medicine, University Hospital Zurich, Zurich, Switzerland; ^2^Medical Faculty, University of Zurich, Zurich, Switzerland; ^3^Department of Experimental Psychology, University of Oxford, Oxford, United Kingdom

**Keywords:** emotion transfer, emotion regulation, empathy, stress, well-being, health, resilience, physicians

## Abstract

Physicians experience many emotionally challenging situations in their professional lives, influencing their emotional state through emotion contagion or social appraisal processes. Successful emotion regulation is crucial to sustain health, enable well-being, foster resilience, and prevent burnout or compassion fatigue. Despite the alarmingly high rate of stress-related disorders in physicians, affecting not only physician well-being, but also outcomes such as physician performance, quality of care, or patient satisfaction, research on how to deal with emotionally challenging situations in physicians is lacking. Based on extant literature, the present article proposes a theoretical model depicting emotions, emotion regulation, and empathy-related processes and their relation to well-being in provider-client interactions. This model serves as a basis for future research and interventions aiming at improving physician well-being and professional functioning. As a first step, interviews with 21 psychiatrists were conducted. Results of qualitative and initial quantitative analyses provided detailed descriptions of the model's components confirming its usefulness for detecting mechanisms linking emotion regulation and well-being in psychiatrist-patient interactions. Additionally, results lend preliminary support for the validity of the model, suggesting that successful regulation of emotions (i.e., achieving a desired emotional state) elicited by cyclical transfer processes in provider-client interactions is associated with both short- and long-term well-being and resilience. Furthermore, empathy-related emotions and their regulation seem to be linked to well-being. Based on the results of the present study, a prospective longitudinal study is under preparation, which is intended to inform effective interventions targeting emotion transfer, empathy-related processes, and emotion regulation in physicians' professional lives. The model and results are also potentially applicable to other health care and social services providers.

## Introduction

In the daily routine of a hospital or a private practice, there are many situations that can elicit emotions in physicians. For instance, breaking bad news to a patient is often perceived as stressful by physicians, and is associated with both increased physiological arousal and difficulties in handling resulting emotions such as sorrow, guilt, or the feeling of failure (1–4). Dealing with demanding patients may also evoke feelings of anger, and experiencing their suffering or death may result in sadness or distress. Yet, in order to prevent emotions from interfering with medical decisions, to stay calm and maintain a professional attitude toward the patient, and to provide high quality care, physicians need to carefully regulate such emotions. Emotional involvement could for example disrupt medical objectivity, resulting in poor judgment and leading to a tendency to over-treat patients (5–8). It has also been shown that physicians downregulate their pain whilst watching visual stimuli depicting physically painful situations (9, 10). The authors have argued that this downregulation is necessary in order to be able to perform painful medical treatments. Thus, emotional detachment has long been a desired emotional state for physicians (5, 11). Through role models and clinical practice, medical students and residents learn to suppress their emotions. In fact, several studies have reported a decline in empathy during medical school and residency [(12, 13); but there are also contradictory findings, see (14–18)]. Furthermore, studies have shown that physicians tend to respond in an informative and biomedical rather than an emotional manner to their patients (19, 20).

However, emotional engagement and especially empathy is of great importance to the patient and to clinical outcomes, as it has been associated with clinical competence and performance [e.g., (21–24)], as well as with the quality of the physician-patient relationship: Patients with an empathetic physician have reported more illness-specific information and concerns, improving diagnostic accuracy (25–28). Physician empathy has also been found to increase patients' participation in, adherence to, and satisfaction with treatment, thus rendering it more effective (26–29). Moreover, empathy has been associated with greater patient enablement, improved communication skills, better decision making, better disease management, better health, less anxiety, and higher quality of life in patients (25, 26, 28, 30–33). In two randomized controlled trials, physician empathy has even been shown to increase patients' immune responses, and to shorten the duration and lessen the severity of a common cold (34, 35). It is not only the patients and the health care system that may benefit from physician empathy, but also the physicians themselves: Empathy has been associated with health, compassion satisfaction, and quality of life, although the causal direction is not yet clear (36–38).

Yet, in their extreme forms, both emotional detachment and emotional engagement take their toll. Emotional over-involvement and exposure to high levels of negative emotions can lead to personal distress, compassion fatigue, emotional exhaustion, and burnout (36, 39–41). For example, it has been shown that the intensity of physicians' regret over difficult patient situations is associated with poor self-rated health, and that adaptive emotion-focused coping strategies protect against the effects of healthcare-related regrets (42). Over-involvement might also result in personal sacrifices such as neglecting one's personal time, hobbies, and family obligations in order to help the patient (8). Personal detachment and unexamined feelings on the other hand may lead to a loss of a professional sense of meaning, objectification of patients, or cynicism, contributing to burnout and depression (5, 6, 43). Therefore, several researchers have highlighted the importance of finding a balance between emotional involvement and emotional detachment (5, 11, 25).

Thus, research has shown that emotionally challenging situations in combination with maladaptive coping strategies can have deleterious effects for physicians, patients, and the health care system. Compared to the general population, stress-related disorders including burnout, depression, substance abuse, and suicide are considerably more prevalent in physicians. Emotionally charged social interactions are thought to be one of the reasons for the alarmingly high prevalence of these disorders. In turn, diminished physician well-being has negative consequences for several clinical outcomes such as physician performance, quality of care, patient satisfaction, and treatment adherence (44–48). These findings further emphasize the importance of facilitating physicians' selection of strategies to cope with emotionally challenging situations in order to enhance their well-being and foster resilience.

However, studies on these topics are scarce. Qualitative studies from the US have examined the types of emotions that clinicians experience, how they manage these emotions, and how these emotions affect the care they provide (49–52). Their results have shown for example that interactions with patients trigger both unpleasant (e.g., anxiety, sadness, frustration) and pleasant emotions (e.g., happiness) which affect the perceived provision of care. Emotion management involves strategies such as self-care (e.g., distraction, relaxation) or seeking social support (e.g., consulting colleagues). However, these findings have not yet been linked to outcomes such as emotional distress, burnout, and well-being. Emotional labor [regulation of emotions or emotional expression to display professionally desired emotions, e.g., (53)] has been associated with lower job satisfaction and adverse health consequences such as stress and burnout in health care personnel, including physicians (54–58). There are also cross-sectional studies, showing that self-reported emotional intelligence in physicians (i.e., the ability to perceive, integrate, understand, and regulate emotions to promote personal growth) is related to higher job satisfaction (57, 59). Moreover, the capacity to self-regulate (emotions, behavior, and other aspects) has been associated with physician well-being (60). However, because these studies used global construct measurements based on self-report rather than behavior, they may only predict the outcomes of emotion regulation in clinical settings to a limited extent. Apart from these methodological issues, the mechanisms linking emotion regulation with positive physician outcomes have not been assessed.

Because empathy is considered a core variable in high-quality patient care, studies linking empathy to burnout and well-being are more common. However, there is no consensus on the definition of empathy, and the causal direction of the relationship between burnout and empathy remains unclear (5, 61, 62). Several approaches to enhancing empathy and interpersonal or communication skills in physicians have been developed, and such programmes have been shown to be effective (63–65). However, as empathy can have negative effects on physician well-being, these programmes might potentially also have harmful consequences. This further highlights the need to examine the mechanisms linking empathy-related processes to negative outcomes.

In order to achieve a better understanding of these mechanisms and thereby facilitate resilience and well-being among physicians, research on how to regulate emotions, on how to be empathic without increasing the risk of vicarious distress, and ultimately on how to find the right balance between emotional involvement and detachment is needed. Here, we aim to contribute to that goal by establishing a theoretical model for understanding emotions and emotion regulation in provider-client interactions. This model can potentially be applied not only to physicians, but also to other professionals working in health care or social services, such as psychologists, nurses, or pastors.

After discussing evidence relating to emotions, emotion regulation, and empathy in the extant literature, a model based on this evidence will be proposed in the following section, which will then be tested and discussed in a qualitative study with additional quantitative data conducted on a sample of psychiatrists.

## Theory of emotions, emotion regulation, and well-being

### Emotions and emotion transfer

Emotions are elicited by external or internal events and have behavioral and neurobiological correlates, allowing a coordinated reaction to the emotion-evoking event spanning experience, behavior, and physiology (66, 67). Consider for example a physician performing a difficult surgical operation. If the surgery threatens to fail (external event) or if the physician anticipates the consequences of failure (internal event), the physician might feel fear or stress [i.e., a subset of negative emotions, see (68, 69)]. This in turn can lead to the physician increasing his or her effort levels (behavior) and releasing additional adrenaline, thereby becoming more alert and prepared for potential complications (neurobiological correlate).

There are several ways in which the social environment can influence and be influenced by emotions. When interacting with an angry patient, the physician might infer from the patient's anger that the treatment is not working as it should, which could then lead to anger about treatment progress on the side of the physician (appraisal 1). Or the physician might perceive the patient's anger as a sign of the patient's inability to cope with the situation and feel sorry for the patient (appraisal 2). The patient's anger might also elicit guilt if the physician thinks that the patient is angry because he or she made a mistake during treatment (appraisal 3). The physician's expression of his or her emotional response may in turn affect the patient, eliciting for example even more anger in the patient, if he or she does not feel understood.

In their work on interpersonal emotion transfer, Parkinson et al. differentiate two basic ways in which the expression of emotions can influence other people [e.g., (70–74); for similar conceptions, see (75–77)]. The first way is through social appraisal processes: Other people's emotions can serve as a source of information regarding an object in the shared environment (as in the example with appraisal 1). Parkinson et al. call this *object-directed transfer*. Others' emotions can also serve as a source of information about the other person (appraisal 2), denoted as *person-directed transfer*. In addition, we argue for a *self-directed transfer*, where an individual uses the emotion of another person as a source of information about himself or herself (as in appraisal 3). These transfer processes need not depend on explicit interpretation but can happen unconsciously, e.g., through conditioning.

A second way in which emotions can be transferred is through emotion contagion. Feelings can spread to another person who then feels the same or a very similar emotion. Through mimicry (matching expression) or mirror representations in the brain (mirror neurons) of what one sees or hears, one can catch the observed emotion. These processes are mostly automatic and non-conscious [e.g., (70–74)]. In contrast to an emotion elicited by conscious or unconscious social appraisal processes, one caught through mimicry or mirror representations need not depend on prior cognitive evaluation or be directed at any specific target (object, person, self). However, it can still influence our appraisal of the shared environment and thereby affect our reaction (71). Parkinson et al. call this *non-directed transfer*.

The transfer of emotions lies at the core of empathy. Even though there is no consensus on how to define empathy, or for that matter sympathy and compassion, a growing body of research concludes that there are at least two components: an affective component (sharing of another's emotions, as in emotion contagion) and a cognitive component (understanding of how another person feels, which can elicit an emotion such as feeling sorry, as in social appraisal). Research in social neuroscience suggests that these processes act in concert and should not be considered in isolation (78–82). For clarity's sake and because the processes related to empathy (as well as to sympathy or compassion) seem to be covered sufficiently by Parkinson et al. conception, our proposed model will not treat empathy-related processes separately, but as forms of interpersonal emotion transfer.

Emotions can help us to respond adaptively to a situation. In a social environment, emotions play an important role regarding affiliation, and social functioning. For example, emotion transfer allows us to bond with other people, to understand their reactions and modulate ours, to pursue shared goals, and to act prosocially. However, there is also a downside to this, namely when emotion transfer impedes social processes such as relational goal pursuit, or when one is over-exposed to negative emotions from others (75, 76, 83). Therefore, it is crucial to regulate emotions.

Emotion regulation itself can also have a social dimension. We can regulate our own emotions to modulate their influence on others. In addition, we can regulate others' emotions (interpersonal emotion regulation), which affects the other person and ourselves in return (70, 84).

### Emotion regulation strategies and abilities

People may use many different strategies to regulate emotions (including stress-related emotions), and these strategies can be classified in many different ways [e.g., (85–87)]. Parkinson and Totterdell, for example, differentiate between cognitive and behavioral strategies, which might either involve some form of diversion (avoiding the situation or the emotion) or engagement [addressing the situation or the emotion, (88)]. Research has shown that some strategies tend to be either adaptive or maladaptive, especially if used dispositionally [e.g., (66, 89)].

However, as Gross (66, 67) and Aldao et al. [e.g., (90)] have pointed out, searching for the optimal strategy regardless of circumstances is misleading. Rather, it might be the flexibility to blend, change, and use strategies in a situationally appropriate way, and the range of available strategies, that are at the core of successful emotion regulation (67, 90, 91). Importantly, it is not always necessary to change emotions in order to respond adaptively to a situation. Acceptance, tolerance, and willingness to approach an emotion without changing it may be equally important in handling emotions. These and other factors such as emotional awareness, beliefs about the mutability of emotions (emotion regulation self-efficacy), or skills covered by the concept of emotional intelligence (92) might help to regulate emotions (90, 93–96). Therefore, researchers now differentiate between emotion regulation strategies (e.g., avoiding the emotion-eliciting situation) and emotion regulation abilities [e.g., variability of the repertoire, flexibility, tolerance, emotional awareness, emotion regulation self-efficacy, (86, 90)].

Research on emotion regulation strategies and abilities underlines the importance of the person-situation-strategy interaction in the success or failure of emotion regulation. This interaction is at the core of the personalized emotion regulation framework proposed by Doré et al. (97). In this framework, features of the person (such as his or her emotion regulation abilities, emotional reactivity, personality, motivations, developmental stage, or biology) interact with features of the situation (such as the type and intensity of the emotion, the modifiability, controllability, and social context of the situation) and with features of the strategy (such as its implicit or explicit deployment, the temporal stage at which it is deployed, the demands a strategy poses, or implementation circumstances), and determine the short-term success of emotion regulation (i.e., whether and to what extent it brings about a desired emotional outcome) but also longer-term well-being.

### Emotion regulation success, well-being, and resilience

In our view, successful emotion regulation in itself (which we understand as the attainment of a desired emotional state through emotion regulation) is important for but does not guarantee optimized well-being. For example, achieving the goal of detachment from an unpleasant emotional situation may bring longer-term costs for the regulator (e.g., depersonalisation). By contrast, facing an unpleasant situation may serve longer-term needs. Although experiencing generally more pleasant and less unpleasant emotions is usually associated with well-being, research suggests that having contextually useful or desired emotions, even if they are perceived as unpleasant, may likewise improve individual functioning and well-being [e.g., (98, 99)]. Therefore, in addition to successful emotion regulation, it is important to balance the needs of maintaining current well-being and long-term well-being.

Researchers commonly distinguish two aspects of well-being. First, subjective well-being involves a positive affective and cognitive appraisal of life in the sense of satisfaction with life and the presence of positive affect (hedonic approach). Second, psychological well-being involves positive functioning in the sense of personal growth, self-acceptance, environmental mastery, autonomy, optimism, engagement, positive relationships, and purpose and meaning in life [eudaimonic approach, (100–104)]. Hence, an emotional state enables well-being when it allows a positive appraisal of life and positive functioning in the current moment and in the long run.

Along the same lines, emotion regulation might be crucial for resilience, i.e., the maintenance of mental health in the face of severe psychological or physical adversity. Resilience has become an important goal in research oriented at preventing stress-related disorders both generally (105) and for physicians and other health care professionals in particular [e.g., (106–108)]. Kalisch et al. see resilience as involving “a dynamic process of adaption to the given stressful life circumstances” [(105), p. 786]. According to this formulation, emotion regulation in a stressful situation is one example of a resilience process. In light of this definition and especially with regard to emotion regulation deficits as transdiagnostic markers in psychiatric disorders (109, 110), emotion regulation may foster resilience and thereby help to prevent stress-related disorders and enhance mental health.

### Theoretical model for emotions and emotion regulation in provider-client interactions

**Figure 1:**
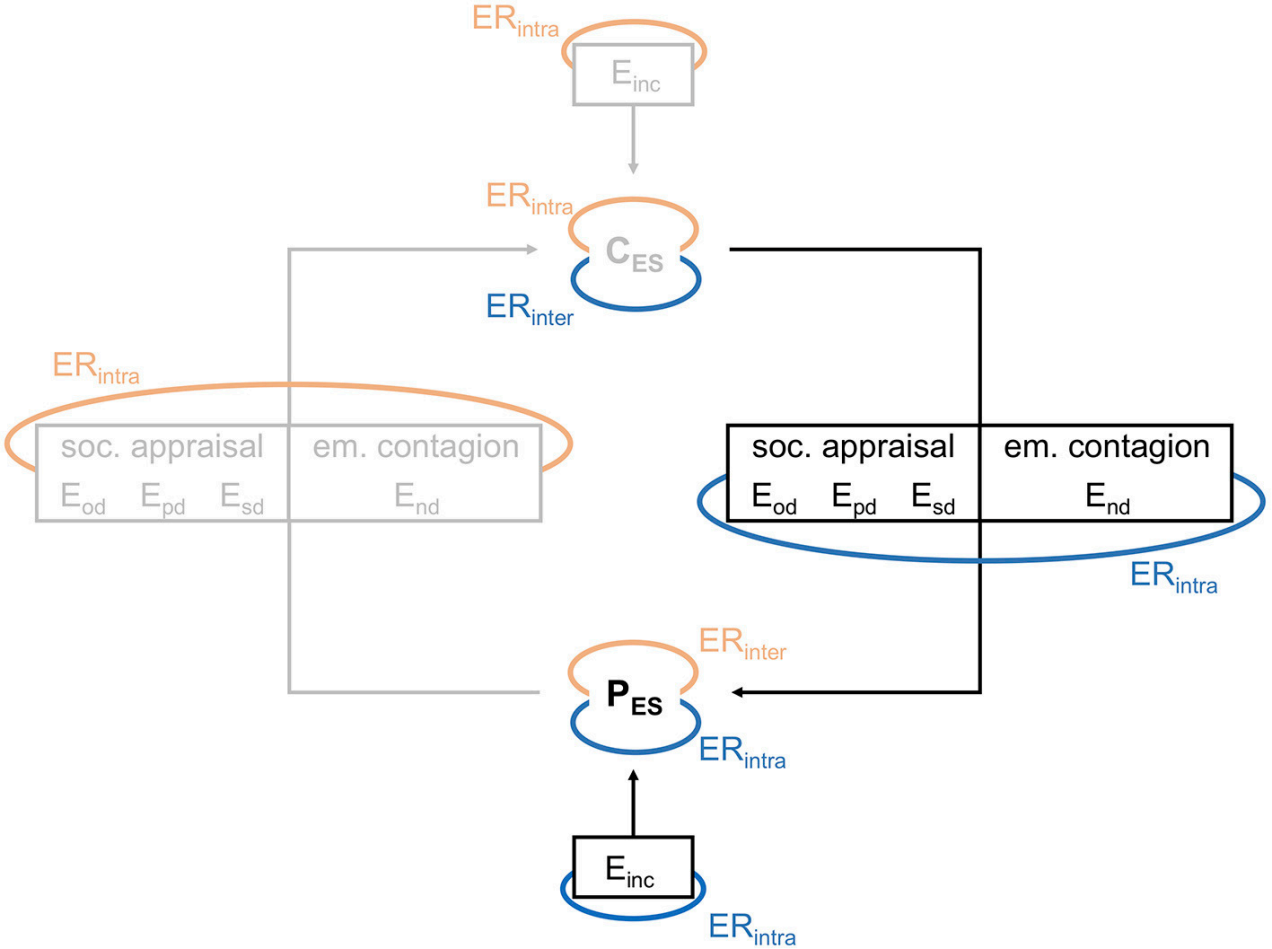
Model for emotions and emotion regulation in provider-client interactions, part 1: Emotion transfer and emotion regulation. P_ES_/C_ES_, emotional state of the provider / client; E_od_, emotions elicited by object-directed interpersonal emotion transfer; E_pd_, emotions elicited by person-directed interpersonal emotion transfer; E_sd_, emotions elicited by self-directed interpersonal emotion transfer; E_nd_, emotions elicited by emotion contagion (non-directed); E_inc_, emotions elicited otherwise (incidental); ER_intra_, intrapersonal emotion regulation; ER_inter_, interpersonal emotion regulation. Blue: provider's emotion regulation. Red: client's emotion regulation.

Based on the discussed literature, we propose the following model for assessing emotions and emotion regulation in provider-client interactions:

When a provider and a client interact with each other, both bring their own emotions, including those elicited by internal or external events related or unrelated to the interaction, and those based on more general moods (referred to as *incidental emotions*, E_inc_). While interacting with each other, both get affected by the other's emotions through social appraisal processes and emotion contagion. This may result in emotions elicited by *object-directed* (E_od_), *person-directed* (E_pd_), *self-directed* (E_sd_), and *non-directed* (E_nd_) interpersonal emotion transfer. All of the resulting emotions contribute to the *emotional state* of the provider (P_ES_) and the client (C_ES_) (Figure 1).

Specific emotions and emotional state more generally can also be influenced by emotion regulation. For example, the provider can influence the degree to which emotion transfer takes place or its selectivity, or can mitigate or enhance already transferred emotions (whereby an emotional state characterized by exclusively downregulated emotions corresponds to emotional detachment and upregulated or unregulated intense emotions to emotional over-involvement). The failure to regulate emotion transfer successfully can result in potentially harmful emotional states, such as having high levels of unpleasant emotions, or emotions which are not useful or desired in a given situation. Also, some kinds of emotion directedness may be more harmful than others [e.g., more self-directed than situation-directed unpleasant emotions, as suggested by Abramson et al. reformulated learned helplessness theory, (111)]. However, transferred emotions might not pose a risk factor at all if regulated (i.e., influenced or embraced) successfully, regardless of their directedness.

The provider (and conversely also the client) can aim to regulate his or her own emotions (referred to as *intrapersonal emotion regulation*, ER_intra_), the client's emotions (*interpersonal emotion regulation*, ER_inter_), or both. Regulating his or her own emotions changes the provider's emotional state and thus the client's as well. Regulating the client's emotions changes the client's emotional state, which in turn influence the emotional state of the provider.

The client may also be directly affected by the emotion regulation strategies of the provider (e.g., the provider may avoid looking at the client to prevent being affected by emotions, which in turn may be disturbing for the client). It is also important to note that the regulation strategies that are deployed by either party do not necessarily affect both parties positively. They might serve the interests of one party, but fail to influence or negatively influence the other party. For example, the provider may downregulate his or her compassion to prevent emotional over-involvement but thereby give the client the impression of being cold and disinterested.

The elicitation and regulation of emotions is thus a cyclical process [see (112)], where provider and client constantly influence each other.

**Figure 2:**
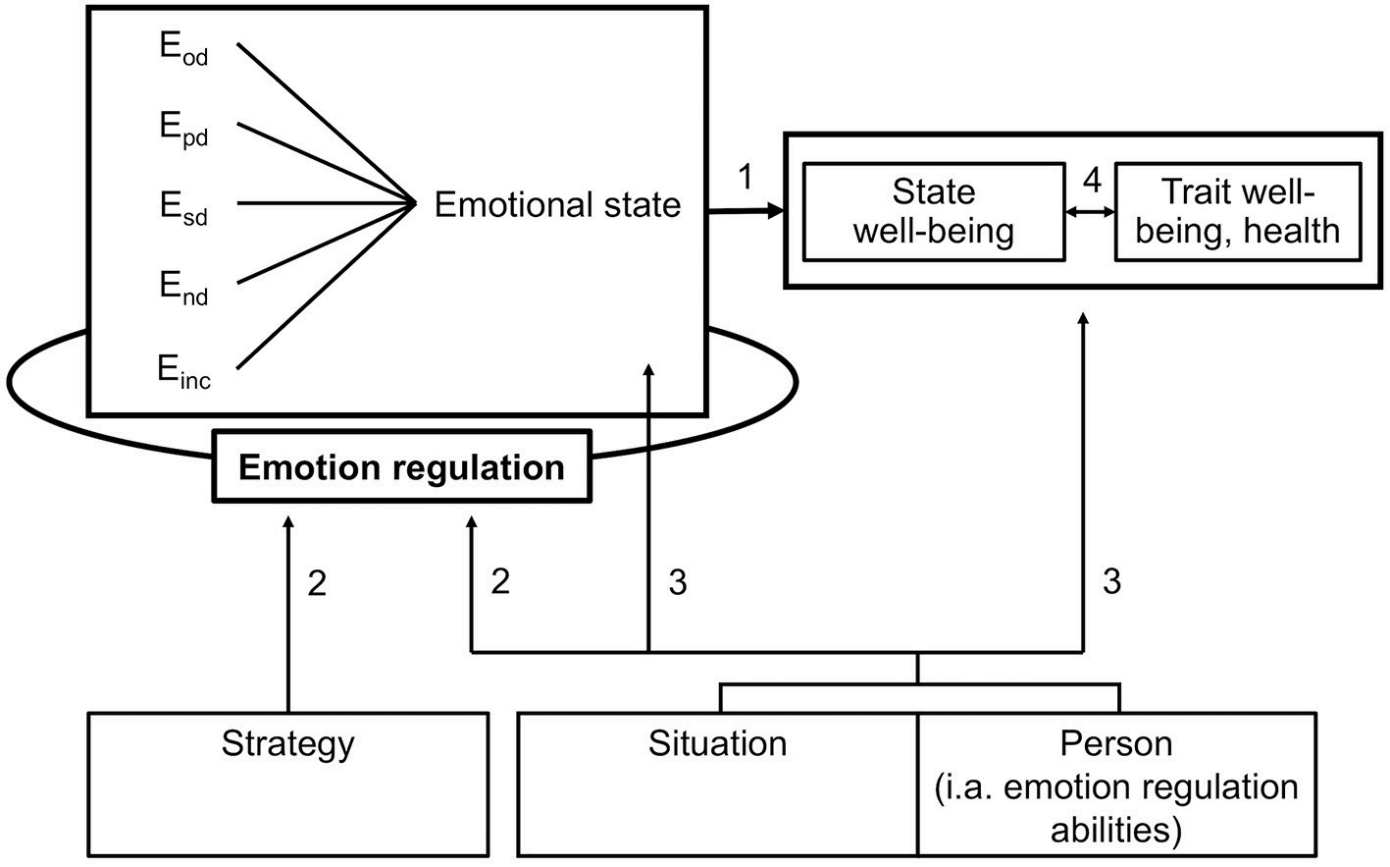
Model for emotions and emotion regulation in provider-client interactions, part 2: Emotion regulation, well-being, and influencing factors. E_od_, emotions elicited by object-directed interpersonal emotion transfer; E_pd_, emotions elicited by person-directed interpersonal emotion transfer; E_sd_, emotions elicited by self-directed interpersonal emotion transfer; E_nd_, emotions elicited by emotion contagion (non-directed); E_inc_, emotions elicited otherwise (incidental).

It is important to note that the emotional state of both parties is continuously changing, consisting of constantly varying emotions and being constantly influenced by emotion regulation. Even if the emotional state is regulated successfully, well-being may be enhanced only in the short run (i.e., state well-being), in the long run (i.e., trait well-being), or not at all. The emotional state is optimally regulated if regulation is successful (i.e., if the desired emotional state is achieved) and enables a balanced maintenance of both state well-being and trait well-being as well as health (Figure 2, path 1).

Emotion regulation itself is influenced by features of the strategy (e.g., tactics applied, energy demands), the situation (e.g., course of the interaction, presence of other people) and the provider (e.g., emotion regulation abilities, path 2). The situation and the personal abilities and traits of the provider influence his or her emotional state as well as his or her state or trait well-being and health in their own right (not only via emotion regulation, path 3). Moreover, state and trait well-being influence each other, as state well-being in the long run contributes to trait well-being and trait well-being shapes the experience of the present moment (path 4).

### Hypotheses

We propose the following hypotheses based on the theoretical model:

1. The ratio between unpleasant and pleasant emotional activation is negatively associated with state and trait well-being. These associations are moderated by emotion regulation (path 1).
2. A lower level of self-perceived useful or desired emotions is negatively associated with state and trait well-being. These associations are moderated by emotion regulation (path 1).
3. Different kinds of emotion directedness (object-, person-, self-, and non-directed emotions) are differently associated with state and trait well-being. These associations are moderated by emotion regulation (exploratory hypothesis, path 1).
4. Successful emotion regulation (i.e., achieving the desired emotional state) is positively related to state and trait well-being and negatively related to indicators of impaired physical and/or mental health. However, we expect these relationships to be of medium size, since emotion regulation success does not guarantee well-being (path 1).
5. Different kinds of emotion regulation strategies are differently related to successful emotion regulation (exploratory hypothesis, path 2).
6. Emotion regulation abilities are positively related to successful emotion regulation (path 2).
7. Emotion regulation abilities are positively related to state well-being, trait well-being, and health, and negatively related to indicators of impaired physical and/or mental health (path 3).
8. State well-being is positively related to trait well-being and health, and negatively related to indicators of impaired physical and/or mental health (path 4).

In order to accurately describe, test, and if necessary revise the assumptions and proposed hypotheses of this theoretical model for research on provider-client and especially physician-patient interactions, a series of qualitative and quantitative studies are currently being prepared and conducted. In the following section, we present data from our first study.

## Empirical support

We conducted in-depth qualitative interviews with psychiatrists and psychiatric residents, each lasting approximately 2 h. We chose psychiatrists because of their insights into, and sensitivity to, their own emotional processes, resulting from their specialist training. The main aim of these interviews was to establish a basis for describing the model more accurately, but also to obtain preliminary evidence regarding the proposed hypotheses, using a series of quantitative questionnaires to assess well-being, emotion regulation abilities, and mental health. Due to the small sample size, we did not test hypothesis 2 or 5, or the moderation effects predicted as part of hypotheses 1 to 3. We also restricted our measurements of (impaired) physical and/or mental health to indicators of burnout, depression, and anxiety. In addition, we measured perceived stress over the past month as an indicator of a prolonged state of unpleasant emotions, which was hypothesized to be negatively related to trait well-being and positively related to burnout, depression, and anxiety. As confirmed by the local ethics committee in Zurich, ethical authorisation was not required, as this study does not fall within the scope of the Swiss Human Research Act. Nevertheless, this study was carried out in accordance with the Swiss Human Research Ordinance (i.e., under strict confidentiality and privacy, with coding of health-related personal data). All participants received written and oral information on the nature, purpose and procedure of the project (especially on potential risks), their right to withhold or revoke their consent at any time, or their right to receive information, and gave written informed consent in accordance with the Declaration of Helsinki.

### Material and methods

#### Participants

By spreading the word, 21 psychiatrists and psychiatric residents were consecutively recruited from several psychiatric hospitals and private practices in the German-speaking part of Switzerland. There were no eligibility criteria in terms of educational stage, therapeutic orientation, or job position. Sample characteristics are listed in Table 1. Interviewees were between 28 and 73 years old (*M* = 51.15, *SD* = 13.12), and the extent of their professional experience ranged from 1 to 45 years (*M* = 19.57, *SD* = 13.41).

**Table 1:** Sample characteristics.

	***n***
Total	21
Women	11
Men	10
Psychiatrists	16
Psychiatric residents	5
Working in a psychiatric hospital	11
Working in a private practice	10
**THERAPEUTIC ORIENTATION**	
Psychodynamic	9
Cognitive-behavioral	9
Systemic	5
Other	2

#### Interviews

The interviews took place between March and May 2018. After providing written informed consent, interviewees were asked to recall a psychotherapy session with a highly distressed patient during the last 2 weeks. This allowed us to standardize the emotion-eliciting situation to a certain degree and compare the reactions between interviewees. Three interviewees selected a session further back than 2 weeks (i.e., in the past 1–6 months), as they did not recall a distressed patient in the specified time period. Most of the recalled therapeutic sessions were held with a patient suffering from either depression (*n* = 8), personality disorders (*n* = 6), mostly narcissistic personality disorder, or posttraumatic stress disorder (*n* = 4). Patients' distress was usually due to a break-down or a personal crisis and many patients were perceived as being demanding toward the interviewees.

Together with the interviewer (SW), interviewees retrospectively explored their own emotions and emotion regulation processes during and after the therapeutic session and the influence these had on themselves and on the patient. Interviews involved asking participants four general open-ended questions, which were followed up with specific questions to obtain fuller information if necessary:

1. Which emotions did you experience while interacting with the patient (type, intensity, valence, trigger, target / directedness)?
2. Did you regulate your emotions (i.e., did you do anything to change or influence your emotions)? If yes, how (tactic and its manner of functioning, perceived effectivity, target, time point of deployment)?
3. What kind of emotional state did you want to attain by regulating your emotions? To what extent did you attain this state?
4. Do you think that your emotions and your emotion regulation influenced the patient or the course of the therapy session? If yes, how?

Some follow-up questions requested a rating (see section Measurement of Model Components). Interviewees were also asked to complete standardized questionnaires on state and trait well-being, emotion regulation abilities, stress, burnout, depression, and anxiety (see below for further details). Out of these interview questions and questionnaires, data on the components of the model (Figures 1, 2) were compiled to exemplify and test it. All interviews were audio-taped and coded by SW. Coding followed a predefined procedure, where the directedness of reported emotions and the strategies behind reported emotion regulation tactics were assigned to previously determined categories (see section Measurement of Model Components). Secondary codings were undertaken by CC except for four interviews, which only allowed for partial coding, due to technical issues (i.e., audio recorder break down). Also, one interview was used to train coding. For each reported emotion or tactic, coding resulted in a pattern of categories. Patterns were compared, and differences discussed and resolved. Interviewees received a small gift (chocolate, worth CHF 20) as compensation for participating.

#### Measurement of model components

##### Emotions and emotional activation

Interviewees described their emotions during the therapeutic session. They rated the intensity of each emotion from 1 = “very low” to 10 = “very high,” and indicated whether it was pleasant, unpleasant, or ambivalent (pleasant and unpleasant or neither pleasant nor unpleasant). A sum score for intensity ratings of pleasant and unpleasant emotions was calculated, indicating the levels of pleasant and unpleasant emotional activation. Interviewees were also asked to indicate the trigger and target of each emotion, which was coded by SW and CC according to the theoretical model (object-directed, person-directed, self-directed, emotion contagion, incidental emotion, other emotion type or direction not indicated by the model as depicted in Figure 1). Congruence between raters was excellent with κ = 0.93 (186 observations), using Cohen's Kappa unweighted.

##### Stress

The level of stress as an unpleasant emotional state in the past month was assessed by the 10-item *Perceived Stress Scale* [PSS-10, (113), validated German version by Klein et al. (114)]. The PSS-10 measures how often one feels overwhelmed and unable to cope with stressors on a 5-point Likert scale (0 = “never” to 4 = “very often”). All items were summed together to yield a total score, with higher scores indicating higher levels of stress. Cronbach's alpha was 0.892.

##### Emotion regulation success

Interviewees described the emotional state they wished to achieve through emotion regulation, and the degree to which they achieved this desired emotional state during and after the therapeutic session as well as later in the evening (1 = “not at all,” 10 = “fully”). These scores served as an indicator of emotion regulation success, with higher scores indicating more successful emotion regulation.

##### Emotion regulation strategies and their effectiveness

Interviewees described tactics they had used to regulate their emotions during and after the therapeutic session and rated the effectiveness of each tactic from 1 = “very low” to 10 = “very high.” A score for the mean effectiveness of all tactics was calculated, with higher scores indicating higher effectiveness. The strategies behind each tactic were coded by SW and CC according to Parkinson and Totterdell's classification (83) of affect regulation strategies (cognitive or behavioral avoidance, distraction by means of thinking (cognitive) or doing (behavioral) something relaxing, pleasant, or demanding, cognitive or behavioral problem solving, reappraisal, vent feelings, social support, other strategy). Congruence between raters was substantial with κ = 0.70 (94 observations), using Cohen's Kappa unweighted.

##### Emotion regulation abilities

Abilities to regulate emotions were measured by the 27 items of the prolonged state version (“within the past 2 weeks…”) of the *Emotion Regulation Skills Questionnaire* [ERSQ, a German questionnaire devised by Berking and Znoj, (94)]. This questionnaire measures abilities such as the awareness, identification, understanding, and acceptance and tolerance of emotions, the perceived ability to modify emotions, the willingness to confront distressing situations, or self-support on a 4-point Likert scale (1 = “not at all”, 4 = “almost always”). Mean scores across all 27 items were calculated, with higher scores indicating higher abilities. Cronbach's alpha was 0.776.

##### State well-being

To assess whether the emotional state immediately after the session enabled state well-being, two questionnaires were used: First, the *Flourishing Scale* [FS, (115), validated German version by Esch et al. (116)], which measures psychological well-being (meaning, positive relations, engagement, social contribution, mastery, self-acceptance, optimism) with 8 items on a 7-point Likert scale (1 = “strongly disagree” to 7 = “strongly agree”). Second, the three subjective well-being scales (life satisfaction, positive feelings, negative feelings) from the *Comprehensive Inventory of Thriving* [CIT, (104), validated German version by Hausler et al. (117)] with 3 items for each scale which were answered on a 5-point Likert scale (1 = “strongly disagree” to 5 = “strongly agree”). Items of the CIT were slightly adapted to fit the purpose of measuring a specific state in the past (i.e., the formulation “most of the time” in some of the items was omitted). Interviewees indicated to what degree the items of the FS and CIT scales were true for their emotional state immediately after the session. A total score for the FS and a mean score for the CIT scales were calculated for each interviewee, with higher scores indicating higher state psychological and subjective well-being. Cronbach's alpha was 0.891 for the FS scale and 0.894 for the CIT scale.

##### Trait well-being

Again, the FS and the subjective well-being scales of the CIT were used. This time, interviewees indicated their agreement with the items in general. Cronbach's alpha was 0.900 for the FS scale and 0.872 for the CIT scale. All participants completed the state version of the well-being questionnaires during the interview and the trait version afterwards.

##### Indicators of mental disorders

Burnout risk was assessed by the *Maslach Burnout Inventory—General Survey* [MBI-GS, (118), German translation by von Känel, 2016] with 16 items on the scales emotional exhaustion, depersonalization, and personal accomplishments rated on a 7-point Likert scale (0 = “daily,” 6 = “never”). Mean scores were calculated, with higher scores indicating higher burnout risk. Depressive symptoms were assessed by the 9-items depression module of the *Patient Health Questionnaire* [PHQ-9, (119), translated by Löwe, 2015] and anxiety by the 7-items *Generalized Anxiety Disorder Scale* [GAD-7, (120), translated by Löwe, 2015] from the PHQ on a 4-point Likert scale (0 = “not at all,” 3 = “almost every day”). Scores were summed together with higher scores indicating higher depressivity and anxiety. Cronbach's alpha was 0.812 for the MBI, 0.483 for the PHQ-9, and 0.785 for the GAD-7.

#### Statistical analysis

Due to the small sample size and the fact that variables were not normally distributed, nonparametric tests (i.e., Spearman correlations and Wilcoxon tests with corresponding effect sizes *r*) were conducted. Because the directions of the expected associations between variables were predefined, one-tailed correlations were calculated for all hypotheses except for hypothesis 3, which was exploratory and therefore tested with two-tailed correlations. Because one interviewee did not experience pleasant emotions, hypothesis 1 and parts of hypothesis 3 were calculated with a sample size of *n* = 20. The same applies to parts of hypotheses 4 and 6, because one interviewee could not rate emotion regulation success in the evening. We did not correct for multiple comparisons to avoid the possibility that truly important associations are deemed statistically non-significant, also given limited power of our analyses. Statistical analyses were conducted with IBM SPSS Statistics, Version 25.

### Results

#### Emotions during psychiatrist-patient interactions

Interviewees reported between 5 and 27 emotions which were mostly pleasant or unpleasant, except for a few cases (*n* = 7) where they were ambivalent or neither pleasant nor unpleasant. The number of pleasant and unpleasant emotions and the extent of pleasant and unpleasant emotional activation is presented in Table 2. Interviewees experienced significantly more unpleasant than pleasant emotions (*z* = −0.2.967, *p* = 0.001, *r* = 0.66) and their unpleasant emotional activation as measured by sum scores of emotion intensity ratings was significantly higher than scores for pleasant emotional activation (*z* = −2.203, *p* = 0.013, *r* = 0.49).

**Table 2:** Number of emotions and extent of emotional activation.

	**Mean**	***SD***	**Range**
Number of pleasant emotions	4.19	2.84	0–10
Pleasant emotion intensity (sum score)	26.86	21.37	0–78
Number of unpleasant emotions	7.52	4.15	1–17
Unpleasant emotion intensity (sum score)	43.14	30.12	5–130.5

*Emotional intensity ratings were summed together for each interviewee, indicating their emotional activation*.

The pleasant and unpleasant emotions reported by interviewees are listed in Table 3, grouped according to Shaver et al. classification (121). All emotions could be assigned to one of the categories of our theoretical model (Figure 1). Among the most frequently reported object-directed emotions were emotions of irritation (e.g., annoyance because of difficult therapy situations or circumstances of the patient), emotions of nervousness (e.g., fear that the therapy might take a bad turn, or uncertainty about how the situation of the patient would develop), and emotions of optimism (e.g., confidence in the therapy or the therapeutic alliance). The most frequently reported person-directed emotions were emotions of affection for the patient (e.g., compassion or benevolence), emotions of irritation (e.g., being annoyed by the patient's behavior), or emotions of nervousness (e.g., being worried that the patient might relapse). By far the most frequently reported emotions were self-directed emotions belonging to the nervousness sub-category. Interviewees reported being stressed or feeling insecure about their own performance. They were also apprehensive that they might lose control over the course of the therapeutic session and their own emotions, or that they might fail in treating the patient. However, they also frequently reported emotions of optimism such as confidence in their skills. The emotions that were most frequently caught by the interviewees concerned the patient's nervousness (tension, helplessness, etc.). Two interviewees reported participating involuntarily in the patient's emotional carousel. Emotions changed over the course of the interaction, with many interviewees reporting having more pleasant emotions toward the end of the therapeutic session as the patient's condition improved.

**Table 3:** Object-directed, person-directed (i.e., patient), self-directed, and non-directed (i.e., emotion contagion) emotions grouped according to the categories and subcategories of Shaver et al. (121).

**Category**	**Sub-category**	**Object-directed Emotions**	***n***
Joy	Cheerfulness (2)	*Joy*	2
	Zest (4)	*Excitement, interest, challenge*	3
		*Drive*	1
	Contentment (2)	*Contentment*	1
		*Gratitude*	1
	Optimism (8)	*Confidence*	6
		*Hope*	1
		*Sense of purpose*	1
	Relief (3)	*Relief*	3
Surprise	Surprise (1)	*Astonishment*	1
Anger	Irritation (12)	Annoyance	6
		Disinclination	2
		Impatience, Agitation	2
		Strain	2
	Frustration (3)	Frustration	3
	Rage (1)	*Anger*	1
Sadness	Sadness (7)	Resignation, Futility, Hopelessness	3
		Powerlessness	2
		Despair	2
	Disappointment (6)	Disappointment	4
		Dismay	2
Fear	Nervousness (12)	Fear	4
		Insecurity, Uncertainty	4
		Helplessness	3
		Tension	1
**Category**	**Sub-category**	**Person-directed Emotions**	***n***
Affection	Affection (19)	*Compassion*	9
		*Benevolence*	5
		*Liking*	4
		*Connectedness*	1
Joy	Cheerfulness (2)	*Joy*	2
	Zest (5)	*Curiosity, Interest*	5
	Contentment (1)	*Gratitude*	1
	Pride (1)	*Pride*	1
	Optimism (5)	*Hope*	4
		*Confidence*	1
	Relief (1)	*Relief*	1
Surprise	Surprise (2)	*Astonishment*	2
Anger	Irritation (14)	Annoyance	11
		Disinclination	2
		Impatience	1
	Disgust (2)	Disgust	1
		Disliking	1
Sadness	Sadness (2)	Hopelessness	1
		Despair	1
	Disappointment (5)	Disappointment	3
		Dismay	2
	Sympathy (3)	Pity	3
Fear	Nervousness (10)	Worry	9
		Uncertainty	1
**Category**	**Sub-category**	**Self-directed Emotions**	***n***
Affection	Affection (1)	*Compassion*	1
Joy	Cheerfulness (2)	*Joy*	2
	Contentment (7)	*Contentment*	4
		*Ease*	3
	Pride (4)	*Pride*	3
		*Feeling Flattered*	1
	Optimism (9)	*Confidence*	4
		*Hope*	2
		*Sense Of Purpose*	2
		*Courage*	1
	Relief (4)	*Relief*	4
Anger	Irritation (3)	Annoyance	3
Sadness	Sadness (3)	Despair	1
		Depletion	1
		Futility	1
	Disappointment (2)	Disappointment	2
	Shame (5)	Guilt	3
		Shame	2
	Neglect (4)	Insult	4
	Sympathy (1)	Pity	1
Fear	Nervousness (46)	Tension, Stress	13
		Insecurity, Uncertainty, Doubt	12
		Apprehension	9
		Incompetence, Insufficiency	6
		Overextension, Helplessness	5
		Cluelessness	1
**Category**	**Sub-category**	**Non-directed Emotions**	***n***
Anger	Irritation (2)	Annoyance	2
Sadness	Suffering (4)	Suffering	4
	Sadness (4)	*Sadness*	3
		*Despair*	1
	Shame (2)	Shame	1
		Guilt	1
	Neglect (1)	Insult	1
Fear	Nervousness (8)	Tension	4
		Helplessness	1
		Insufficiency	1
		Doubt	1
		Distress	1

*In order to better fit the data, the category name “love” was replaced by “affection.” Emotions which were reported as being pleasant are in italic. The number in bracket denotes the frequency of reported emotions per sub-category*.

It is important to mention that interviewees used the terms “compassion,” “empathy,” and “sympathy” interchangeably to describe the same phenomena, namely empathy-related processes such as sharing or understanding the patient's distress. Some of the interviewees perceived these processes as pleasant (*n* = 9), some as unpleasant (*n* = 3), and some as ambivalent (*n* = 4). Many of the interviewees reported sharing components (emotion contagion) as being unpleasant, and understanding components (social appraisal) as being pleasant. Apart from empathy-related emotions, other emotions (e.g., anger directed at the situation) were also perceived as unpleasant by some interviewees, while others perceived them as pleasant (usually because it enabled them to better connect with the patient).

In some cases, incidental emotions were reported. For example, the case of one patient triggered a childhood memory in an interviewee, who then felt sadness. Moreover, emotions related to the therapy session were not only reported to be present during the session itself, but also before and after (e.g., anticipation or relief). These emotions are not discussed further here.

#### Emotion regulation

Interviewees reported using between 2 and 10 different strategies to regulate their emotions (*M* = 6.71, *SD* = 2.61) during and after the therapy session. Most of the reported tactics served several strategies at the same time (e.g., speaking to colleagues often served the strategies of cognitive problem solving, venting feelings, and social support). While most emotion regulation tactics served strategies described by Parkinson and Totterdell (88), participants reported some tactics for which strategies had to be added to the original classification. Strategies and examples of corresponding tactics are reported in Table 4.

**Table 4:** Emotion regulation strategies according to Parkinson and Totterdell (88) with examples of corresponding tactics used during (^d^) and after (^a^) therapy sessions from the present interviews (italics), and number of participants who reported having deployed the respective strategy.

	***n***	**Cognitive**	***n***	**Behavioral**
**DIVERSION**				
Disengagement	1	Avoid thinking about the problem	1	Avoid problematic situation *(e.g., leave the room ^*d*^)*
Distraction	8	Think about something pleasant *(e.g., holidays ^*d*^, hobbies ^*d*^, activities later on that day ^*d*^)*	14	Do something pleasant *(e.g., hobbies ^*a*^, every-day activities ^*a*^)*
	3	Think about relaxing thoughts *(e.g., color blue ^*d*^, mountains ^*d*^)*	15	Do something relaxing *(e.g., breathe ^*d*^, calming body exercises ^*a*^, hot shower ^*a*^)*
	0	Think about something that occupies attention	10	Perform a demanding activity *(e.g., hobbies ^*a*^, every-day activities ^*a*^)*
Other^*^			3	Suppress emotions^*^
**ENGAGEMENT**				
	16	Reappraise *(e.g., accept emotions as being legitimate ^*d, a*^, reinterpret situation ^*d, a*^, saying to oneself one did the best one could ^*d, a*^)*	9	Vent feelings *(e.g., playing music ^*a*^, speaking with colleagues ^*a*^, laughing together with patient ^*d*^)*
	5	Think about social support^*^ (e.g., think about help from supervisor ^*d*^)	9	Seek help or comfort from others (social support) *(e.g., speaking to colleagues or spouse ^*a*^, supervision ^*a*^, case review ^*a*^)*
	12	Think about how to solve problem *(e.g., analyzing situation and plan next steps ^*d, a*^)*	15	Take action to solve problem *(e.g., using therapeutic techniques to change the course of the therapeutic session ^*d*^, taking an observer-perspective ^*d*^, change body posture ^*d*^)*
	6	Self-compassion^*^ (e.g., soothe oneself ^*d, a*^)	15	Boundary management* (e.g., setting symbolic boundaries between one's roles as therapist or private person ^*d, a*^, deliberate changing of one's roles ^*d, a*^)

Strategies marked with

* * were added to the original classification*.

A group of tactics deployed by three quarters of the interviewees that could not be assigned to one of the strategies of Parkinson and Totterdell's classification (88) was what we call boundary management. Several interviewees reported that they stepped out of their role as therapist (either intentionally or more implicitly) in order to feel themselves more clearly. They used tactics such as diverting their gaze from the patient and centring awareness on their own body (during the session), changing the room, or performing everyday activities, e.g., watering the plants or drinking coffee (after the session), in order to reconnect with their private selves. Others reported stepping deliberately back into their role as therapist when they felt that they were swept away by feelings, for example by moving their body into an upright position or by using typical therapeutic techniques such as psychoeducation to remind themselves and the patient of their role. Moreover, several interviewees used symbolic boundaries between their private selves and their roles as therapists or between themselves and the patient, such as physical space, doors between the therapy room and their private room, or imaginary walls. When they felt overwhelmed by the patient's emotions and / or their own reactions, they reported setting boundaries e.g., by enlarging physical space or by strengthening imaginary walls between them to gain emotional distance and regulate the transfer of emotions. Even though these tactics are similar to distraction or disengagement, boundary management is distinct from diversion strategies as the goal is not to avoid the situation or emotion, but to actively (dis)connect with one's roles in order to deal with the situation or emotion. Thus, it is an engagement strategy. Since it does not involve addressing an emotion-eliciting situation or emotion itself as in reappraisal and problem-solving, but rather delineates the framework or domain in which emotions are allowed to unfold, we see it as a new, distinct strategy.

Approximately three quarters of the interviewees used the engagement strategies of reappraisal and cognitive and behavioral problem-solving as means of regulating their emotions. A frequently reported behavioral problem-solving tactic characteristic of the therapeutic setting was taking an observer-perspective to distance oneself from the situation, enabling a new and neutral look on one's own emotions and the patient. Reappraisal included acceptance tactics such as deliberately embracing the presence of unpleasant emotions as being legitimate or important (and other related processes). Imagined social support (e.g., thinking about helpful others or about what helpful others would say) or real social support were used by half of the interviewees. Frequently used diversion strategies were behavioral distraction by doing something pleasant, relaxing, or demanding, which most often referred to leisure time activities such as sports and hobbies. Distraction strategies typically targeted the emotional state as a whole, whereas other strategies such as reappraisal usually targeted a single emotion or several specific emotions together.

Interviewees rated the mean effectiveness of their tactics as 7.52 (*SD* = 1.25, highest possible score = 10). In some cases, interviewees indicated that tactics they used might also have affected their emotional state in the opposite direction to the intended one, and there were indeed tactics that had positive short-term effects, but no or even negative effects in the long run. For example, one interviewee reported that educating the patient helped him to regain control and security in the situation very effectively, but did not improve his general emotional state. On the contrary, it drained him of energy and had a negative overtone.

Not all tactics were chosen intentionally. Some interviewees reported using opportunities presented to them fortuitously by the situation to regulate their emotions. For example, one interviewee left the room to get a medical device for the patient and used the chance provided by this physical movement to feel herself again. Another interviewee reported being swept away by emotions and took the chance to distance himself and regain his own composure when the patient went to stand by the window. Interviewees also seemed to deploy many tactics automatically, and only became aware of their implementation when reflecting about the session afterwards.

Emotion regulation abilities were rated on average at 3.65 (*SD* = 0.20, highest possible score = 4).

Adjectives most often used to describe the emotional state that interviewees aimed to achieve were “at ease” / “balanced” (*n* = 16), “content” / “cheerful” / “good” (*n* = 14), and “in control” / “competent” (*n* = 4). On average, interviewees achieved their desired emotional state to the extent of 5.62 (*SD* = 2.64) during the therapeutic session, 6.50 (*SD* = 2.06) after the session, and 8.43 (*SD* = 1.73) in the evening, with a highest possible score of 10.

Even though the extent to which interviewees achieved their desired emotional state (i.e., emotion regulation success) improved significantly from the session to immediately afterwards (*z* = −1.941, *p* = 0.027, *r* = 0.42) and from immediately after the session to the evening (*z* = −0.3.220, *p* < 0.001, *r* = 0.72), three interviewees indicated that their emotional state immediately after the session was somewhat lower than it had been during the session. The reason for this decline was that they allowed themselves to have doubts about their performance only after the session and not while it was happening.

All interviewees felt that their own emotions and emotion regulation had an influence on the patient and the session. They used this influence for example by confronting the patient with their own emotions as a therapeutic technique or by regulating their own emotions to display professionally desirable emotions such as tranquility (emotional labor). In many instances, this served as a means of changing the patient's emotional state (interpersonal emotion regulation). One interviewee took the patient for a walk to calm him down and renew his focus, which is an interpersonal emotion regulation strategy. As a consequence this tactic also lifted her own spirit.

#### Well-being and mental health

Scores on state and trait subjective and psychological well-being are reported in Table 5. Scores on state and trait measures of both subjective and psychological well-being differed significantly, with trait scores being higher than state scores (*z* = −3.235, *p* < 0.001, *r* = 0.71 for subjective well-being and *z* = −2.065, *p* = 0.019, *r* = 0.45 for psychological well-being). This indicates that our measures were sensitive to state variations.

**Table 5:** Subjective and psychological well-being scores.

	**State**		**Trait**	
	**Mean**	***SD***	**Mean**	***SD***
Life satisfaction	3.94	0.65	4.13	0.52
Positive affect	3.64	0.98	4.19	0.63
Negative affect (reversed)	3.89	0.90	1.51	0.49
Total subjective well-being	3.82	0.75	4.27	0.45
Psychological well-being	46.93	6.10	48.81	5.00

*Highest possible score for subjective well-being scales is 5, for psychological well-being 56*.

Burnout, anxiety and depression scores are reported in Table 6. Regarding burnout, three interviewees had scores indicating an increased risk for burnout, all others were in the no-risk group. Four interviewees had mild depression scores, all others reported minimal scores. Four interviewees had mild anxiety scores, one had moderate anxiety scores, all others reported minimal scores.

**Table 6:** Mental health scores.

	**Mean**	***SD***
Burnout symptoms	0.91	0.48
Anxiety symptoms	3.76	2.64
Depressive symptoms	3.10	1.79

*Highest possible score for burnout symptoms is 6, for anxiety symptoms 21, and for depressive symptoms 27*.

#### Relations between emotions, emotion regulation, well-being, and mental health

Although sample size and statistical power were low, initial statistical analyses were run to gain preliminary insights into potential relationships between model components.

**Hypothesis 1**. The ratio of unpleasant to pleasant emotional activation (quotient of sum intensity ratings of unpleasant emotions and of pleasant emotions) was significantly negatively associated only with state subjective well-being (ρ = −0.496, *p* = 0.013) and state psychological well-being (ρ = −0.433, *p* = 0.028), but not with trait well-being.

**Hypothesis 2**. was not tested.

**Hypothesis 3**. For pleasant and unpleasant emotions separately, intensity ratings within each emotion direction (object-, person-, self-, and non-directed) were summed together, to yield scores for the emotional activation of each emotion direction. A ratio was calculated by dividing emotional activation of each direction by total pleasant or unpleasant emotional activation. Results showed that higher non-directed unpleasant emotional activation (i.e., more emotion contagion) relative to total unpleasant emotional activation was associated with lower state subjective well-being (ρ = −0.569, *p* = 0.007). By contrast, higher object-directed unpleasant emotional activation relative to total unpleasant emotional activation was related to more positive state psychological well-being (ρ = 0.443, *p* = 0.044). Higher person-directed pleasant emotional activation relative to total pleasant emotional activation was significantly associated with more positive state subjective well-being (ρ = 0.500, *p* = 0.025) and marginally associated with more positive state psychological well-being (ρ = 0.401, *p* = 0.080). Moreover, higher object-directed pleasant emotional activation relative to total pleasant emotional activation was negatively associated with trait subjective well-being (ρ = −0.501, *p* = 0.024).

**Hypothesis 4**. Successful emotion regulation (i.e., the extent to which interviewees achieved their desired emotional state) during the therapeutic session was significantly positively associated with state subjective well-being (ρ = 0.609, *p* = 0.002) and state psychological well-being (ρ = 0.523, *p* = 0.007). Since state well-being was measured in connection with the time point immediately after the session, relations between state well-being and emotion regulation success after the session and in the evening were not tested. Emotion regulation success in the evening was significantly associated with higher trait subjective well-being (ρ = 0.480, *p* = 0.016) and trait psychological well-being (ρ = 0.776, *p* < 0.001). Moreover, emotion regulation success during the session (ρ = −0.394, *p* = 0.038) and after the session (ρ = −0.377, *p* = 0.046) was significantly associated with lower burnout scores, and emotion regulation success in the evening was marginally associated with lower burnout (ρ = −0.317, *p* = 0.087), anxiety (ρ = −0.327, *p* = 0.080), and depression scores (ρ = −0.370, *p* = 0.054).

**Hypothesis 5**. was not tested.

**Hypothesis 6**. Emotion regulation abilities were significantly positively related to emotion regulation success during the session (ρ = 0.385, *p* = 0.042), after the session (ρ = 0.373, *p* = 0.048), and in the evening (ρ = 0.685, *p* < 0.001).

**Hypothesis 7**. Emotion regulation abilities were significantly positively associated with state psychological well-being (ρ = 0.506, *p* = 0.010), trait psychological well-being (ρ = 0.681, *p* < 0.001), and trait subjective well-being (ρ = 0.448, *p* = 0.021). Although the correlations between emotion regulation abilities and burnout, depression, and anxiety were all negative, none of them were statistically significant.

**Hypothesis 8**. State subjective well-being was significantly positively related to trait subjective well-being (ρ = 0.564, *p* = 0.004) and significantly negatively related to burnout (ρ = −0.587, *p* = 0.003). State psychological well-being was significantly positively related to trait psychological well-being (ρ = 0.717, *p* < 0.001), and significantly negatively related to burnout (ρ = −0.579, *p* = 0.003) and depression (ρ = −0.501, *p* = 0.010). All other correlations were in the expected direction, but not significant.

The average level of perceived stress over the past month was 11.14 (*SD* = 5.71, highest possible score = 40). Stress was significantly negatively related to trait subjective well-being (ρ = −0.624, *p* = 0.001) and marginally related to trait psychological well-being (ρ = −0.317, *p* = 0.081), and significantly positively related to anxiety (ρ = 0.615, *p* = 0.002), but not to burnout and depression.

### Discussion

The main aim of this study was to assess whether our theoretical model is applicable to physician-patient interactions and to provide a basis for describing its operation more accurately. Results generally confirm the usefulness of our model for understanding physician-patient interactions. Regarding the first part (Figure 1), all emotions elicited by the interaction could be assigned to the categories specified in the model. Generally, interviewees experienced more unpleasant than pleasant emotions, which comes as no surprise, given that they were asked to focus on a therapeutic interaction with a highly distressed patient. By far the most commonly reported emotions were self-directed and in the category of nervousness, containing emotions such as stress, insecurity, and apprehension regarding the interviewees themselves or their performance. Emotions changed over the course of the therapeutic session, often shifting toward more pleasant emotions as the patient's condition improved. Furthermore, all interviewees believed that their emotions influenced the patient and reported that they used this influence as a therapeutic technique (e.g., being calm in order to calm the patient down). This is strongly in line with the proposed cyclical process of emotion transfer in our model.

Also in line with the model, interviewees regulated their emotions at several stages. Some emotion regulation tactics helped to regulate emotion transfer itself, while others regulated already transferred emotions, either targeting specific emotions or the emotional state as a whole. Interpersonal emotion regulation (i.e., regulation of patients' emotions) was seldomly reported. However, as lending help to regulate emotions is an integral part of psychotherapy, this may not have been interviewees' explicit focus of attention in this specific sample.

Boundary management was one of the strategies that helped in the regulation of emotion transfer, preventing interviewees from becoming emotionally over-involved. Boundary management tactics such as switching between interviewees' roles or preventing emotions from being taken into their private sphere also helped to regulate emotions that had already been transferred. Indeed, boundary management was one of the most frequently used strategies confirming the importance of keeping boundaries for well-being in order to enable or prevent positive and negative spill-over from one domain into the other [e.g., work and home, (122)]. Another strategy that was frequently used to regulate already transferred emotions was reappraisal, which included acceptance of unpleasant emotions as being legitimate or important. Acceptance is one of the core emotion regulation abilities [e.g., (93, 94)] and its importance in reducing stress and enhancing well-being is also evident in the effectiveness of acceptance or mindfulness based interventions [e.g., (123, 124)]. Problem-solving and distraction by doing something pleasant, relaxing, or demanding in terms of leisure time activities were used equally often, mostly to regulate emotional state as a whole. Research has shown that detachment from work and recovery through meaningful off-job activities are particularly important to well-being (122, 125).

The second part of our model (Figure 2) was also supported by the data from the present study. Emotions and emotional state were significantly related to well-being: Having higher unpleasant emotional activation than pleasant emotional activation was associated with lower state well-being. Although trait well-being was not related to emotions experienced in a single session, being repeatedly in emotionally difficult situations seems to be linked with well-being and health in the long run, as indicated by the positive association with chronic stress. These findings point to the importance of regulating emotion transfer and already transferred emotions, enhancing pleasant emotions and embracing or mitigating unpleasant emotions. Indeed, successful emotion regulation (i.e., bringing about a desired emotional state) may protect against the effects of harmful emotional states during the session, as suggested by the positive association between emotion regulation success during the session and state well-being. For long-term well-being, a successfully regulated emotional state in the evening rather than during the day may be crucial, as suggested by the strong association between trait well-being and emotion regulation success in the evening. Importantly, successful emotion regulation was also related to lower burnout scores and, marginally, to lower anxiety and depression scores, indicating a protective effect on mental health.

As proposed by our model (Figure 2), higher emotion regulation abilities such as emotion regulation self-efficacy were related to well-being in the short and in the long run. Higher state well-being was associated with higher trait well-being and less burnout symptoms. Taken together, these results lend preliminary support to the validity of our model as a whole, suggesting that emotions elicited by cyclical transfer processes in provider-client interactions are linked to short-term well-being. Over time, they may accumulate and influence longer-term well-being. It thus seems likely (but needs to be tested by longitudinal studies) that successful emotion regulation is important for maintaining both state and trait well-being and fostering resilience. However, larger sample sizes are needed to confirm these associations.

A striking finding was that object-, person-, self-, and non-directed emotions had different associations with state well-being, indicating that not all pleasant emotions are equally beneficial, and not all unpleasant emotions equally harmful. Despite their preliminary nature, our data provide initial insights into mechanisms that may link emotion transfer and well-being in physicians. Higher patient-directed pleasant emotional activation relative to total pleasant emotional activation seemed to be positively associated with state well-being. Most of the pleasant emotions directed at the patient were empathy-related emotions (i.e., compassion resulting from understanding the patient's distress). Interestingly, experiencing higher non-directed unpleasant emotional activation (i.e., emotion contagion, most often of the patient's distress) was significantly negatively associated with state well-being. This finding underlines the crucial importance of empathy-related emotion transfer for state well-being, suggesting that sharing the patients' emotions may be harmful, whereas feelings based on understanding the patients' emotions may have beneficial effects. Also supporting this finding, interviewees perceived empathy-related emotions based on emotion-sharing as rather unpleasant and empathy-related emotions based on understanding as rather pleasant. Although these interpretations are tentative, they correspond well to other research on empathy-related processes. Recent studies from a social neuroscience perspective clearly highlight the importance of maintaining self-other distinction in empathy-related processes in order to prevent compassion fatigue and sustain well-being. Thus, feeling *with* the other person (i.e., emotion-sharing) is potentially harmful, whereas feeling *for* the other person (i.e, understanding the other's situation) is not [e.g., (81)]. This general principle has also been discussed in relation to physicians [e.g., (36, 62, 80)].

Furthermore, experiencing higher unpleasant emotional activation directed at the situation (object-directed) seems to be positively associated with well-being. Many of the unpleasant emotions directed at the situation involved irritation or nervousness corresponding to the patient's own emotions regarding the situation. These emotions often helped with the process of connecting with the patient, which might explain their positive association with state well-being. Indeed, this again seems to highlight the importance of an emotional bonding with the patient for state well-being. Therefore, empathy-related and similar emotions might prove important mechanisms linking emotions and emotion regulation with well-being.

#### Strengths, limitations, and future research

The interviews were first and foremost qualitative, serving the purpose of describing the model more accurately. In order to be able to describe emotion transfer processes and their effects on interviewees in as much detail as possible, interviews focused on one specific therapeutic session in the last 2 weeks. By contrast to previous studies, which have asked physicians very generally about their emotions and emotion regulation strategies, this design enabled us to record the myriad of emotions elicited by one interaction more exactly, together with their triggers, targets, interconnectedness and effects on physician and patient. Furthermore, we tracked the regulation of these emotions and how regulation affected interviewees' state well-being. The main strength of this study thus lies in the rich description of our model, providing important insights into the outlined processes and highlighting key target variables (e.g., boundary management and the complex interconnectedness of empathy-related emotions) for future research.

However, our interview schedule was informed by theory, resulting in a predominantly top-down approach. Although we used open questions to assess whether there were emotions or emotion regulation strategies that did not fit to our model, there was only limited scope for the interviewees to inform theory (bottom-up) in order to test the content validity of the model.

Further, as interviews were retrospective, our findings may have been affected by recall bias. Indeed, the reconstruction of emotional experiences in the past is known to be biased by several factors, and this fact has stimulated the use of experience sampling methods for assessing emotional experiences in daily life [e.g., (126, 127)]. For example, research demonstrates that memory of emotional episodes draws on the moment of the highest emotion intensity and the end of the episode, as suggested by the peak-and-end rule (128). Thus, as emotions of interviewees have often improved toward the end of the therapeutic session, negative emotional experiences during the session may have been underestimated, just to name one example of a potential bias.

Third, the recalled interaction might not have been representative of the interviewees' typical emotions and emotion regulation during physician-patient interactions and their influence on more general well-being and health. Moreover, these reports might only be generalisable to other therapeutic situations and physicians of other disciplines to a limited extent. Also, it remains unclear whether our model is applicable to more complex physician-patient interactions involving additional people such as the patient's family or other health care personnel. As the literature implies, team climate and senior physicians may exert a particularly important influence on the physician's emotions and emotion regulation (5, 44, 47, 48). Research has so far shown that the same emotional transfer processes (social appraisal and emotion contagion) are at work in groups, resulting in a group affective tone. These processes have been shown to influence social functioning of the group similarly to the one-to-one setting, whereby leaders and followers exert a different influence (129). Such variables are lacking from the model and need at least to be considered as situational variables influencing emotions and emotion regulation (Figure 2, paths 2,3).

Fourth, interviewees could only report on what they had consciously experienced and done. Emotions which had automatically been suppressed, emotions that the interviewees were unaware of, or tactics which had been deployed implicitly may have had substantial effects on the therapists' emotional state, emotion regulation success and well-being, constituting extraneous variables that we could not measure or control.

Interview questions and questionnaires also allowed us to generate quantitative data, providing preliminary evidence regarding the proposed hypotheses. However, the sample size was in many cases too small to detect significant effects. Finally, the cross-sectional design did not allow to assess causal relationships between variables.

Future studies should target a wider range of physician-patient interactions prospectively, using momentary assessments (e.g., psychophysiological measurements) to prevent recall bias and enable the detection of more implicit emotions and emotion regulation. However, before applying the model to such prospective studies, further validation studies are needed, including both qualitative studies of physicians of other specialties (e.g., in primary care disciplines) and quantitative studies with larger sample sizes. Results of the present study indicate that investigating effects of the directedness of emotions might be especially useful for detecting mechanisms linking emotions and emotion regulation to well-being. Furthermore, future studies should examine the effectiveness of emotion regulation strategies, additional emotion regulation abilities such as flexibility and repertoire variability, and the influence of the presence of other people and related situational variables, which we did not assess.

In order to further investigate emotion transfer and emotion regulation in provider-client interactions, validation studies for our model as well as a prospective longitudinal study with experimental and momentary assessments (e.g., psychophysiological measurements) are under preparation. Results may inform effective interventions targeting emotion transfer, empathy-related processes, and emotion regulation on an everyday basis in physicians' professional lives. Moreover, our model and results may also prove to be applicable in other health care and social services contexts.

## Author contributions

SW reviewed the literature, developed the theoretical model, and design of the study, conducted interviews, performed the data analysis, and wrote the manuscript. MP and US contributed substantially to the conception of the model as well as the study design and conduction. CC coded all interviews. BP and RvK provided substantial input on all stages of conception and study design. All authors contributed to manuscript revision, read, and approved the submitted version.

### Conflict of interest statement

US is hosting this Frontiers Research Topic and has collaborated with the guest editor. MP has collaborated with the guest editors in the past five years. The remaining authors declare that the research was conducted in the absence of any commercial or financial relationships that could be construed as a potential conflict of interest.
